# Temporal trends in neurologically intact survival after paediatric bystander-witnessed out-of-hospital cardiac arrest: A nationwide population-based observational study

**DOI:** 10.1016/j.resplu.2021.100104

**Published:** 2021-03-10

**Authors:** Yoshikazu Goto, Akira Funada, Tetsuo Maeda, Yumiko Goto

**Affiliations:** a Department of Emergency and Critical Care Medicine, Kanazawa University Hospital, Takaramachi 13-1, Kanazawa 920-8640, Japan; b Department of Cardiology, Osaka Saiseikai Senri Hospital, Tukumodai 1-1-6, Suita 565-0862, Japan; c Department of Cardiology, Yawata Medical Center, Yawata I 12-7, Komatsu 923-8551, Japan

**Keywords:** Out-of-hospital cardiac arrest, Children, Outcomes, Epidemiology

## Abstract

**Aim:**

Trends in neurologically intact survival after paediatric out-of-hospital cardiac arrest (OHCA) remain unclear. In the present study, we aimed to determine trends in 1-month neurologically intact survival after paediatric OHCA over time.

**Methods:**

We reviewed the data of 5461 children (aged < 18 years) who experienced bystander-witnessed OHCA and were included in the nationwide Japanese registry from 2005 to 2017. Patients were divided into three groups according to study period: 2005–2010, 2011–2015, and 2016–2017. We analysed the trends in 1-month neurologically intact survival rates over time.

**Results:**

The risk-adjusted odds of 1-month neurologically intact survival (odds ratio, 1.86; 95% confidence interval, 1.41–2.44) were significantly improved by 2016–2017 compared with baseline. Similar improvements in 1-month neurologically intact survival rates were observed with both standard bystander cardiopulmonary resuscitation (CPR) with rescue breaths and chest compression-only bystander CPR (*P* for trend < 0.05 and < 0.001, respectively). In the subgroup analyses by aetiology, the 1-month neurologically intact survival rate in patients with OHCA of non-traumatic origin significantly increased from 11.8%–15.1% to 19.7% (*P* for trend < 0.001) but not in those with OHCA of traumatic origin (from 4.9% to 3.4% to 4.1%; *P* for trend = 0.29).

**Conclusion:**

The 1-month neurologically intact survival rate significantly increased from 2005 to 2017 in Japanese children with bystander-witnessed OHCA, regardless of bystander CPR type; This increase was noted in patients with OHCA of non-traumatic origin but not in those with OHCA of traumatic origin.

## Introduction

Early bystander cardiopulmonary resuscitation (CPR) is a critical component in the chain of survival after out-of-hospital cardiac arrest (OHCA).^1^, ^2^ To increase the bystander CPR rate, the current CPR guidelines for children with cardiac arrest recommend chest compression-only CPR (chest-compression-only CPR) for both untrained and trained bystanders unwilling to perform rescue breaths before the arrival of emergency medical service (EMS) personnel.^3^, ^4^ In Japan, emergency dispatch centres have become more active in relaying CPR instructions to bystanders who can then perform CPR since the updated American Heart Association guidelines 2000.^5^ In 2006, dispatcher-assisted CPR instructions were revised from CPR with rescue breaths (standard CPR) to chest-compression-only CPR.^6^ Therefore, the chest-compression-only CPR rate significantly increased in Japan, accounting for approximately 70% of all CPRs.^6^ Following the 2010 and 2015 updates to the CPR guidelines, the sequence in performing CPR has changed from ‘airway–breathing–circulation’ to ‘circulation–airway–breathing’.^7^, ^8^ Moreover, the application of paediatric targeted temperature management as part of post-resuscitation care may contribute to the increase in favourable outcomes following the updates in the CPR guidelines.^9^ In adults with OHCA, any type of CPR was associated with a two-fold increase in survival rates compared with no CPR.^10^ In the United States (US), the survival rates of children with in-hospital cardiac arrest have significantly improved.^11^ However, previous studies reported conflicting results regarding the trend in survival rates of children with OHCA.^12^, ^13^ One study showed no improvement in survival after OHCA of cardiac origin.^12^ Meanwhile, another study showed an improvement in the rate of survival to hospital discharge among paediatric patients with non-traumatic OHCA.^13^ During the periods in which various guidelines were adopted, temporal trends in survival and neurologically intact survival according to bystander CPR type and aetiology have not been reported in paediatric patients with OHCA.

In this study, we aimed to determine the change in outcomes after paediatric bystander-witnessed OHCA over time according to the CPR type and OHCA cause using the Japanese nationwide registry data.

## Methods

### Study design and setting

This nationwide, population-based observational study included all paediatric patients (aged < 18 years) who experienced bystander-witnessed OHCA and were resuscitated by EMS personnel in Japan between January 1, 2005 and December 31, 2017. Patients were excluded if they (1) were aged ≥ 18 years, (2) did not receive resuscitation from an EMS personnel, (3) had no witnesses to their cardiac arrest, (4) had only EMS personnel as witnesses, (5) received rescue breathing-only bystander CPR or AED-only bystander intervention, or (6) had unknown outcomes or age. The study was approved by the institutional review board of Kanazawa University (No. 1263-8). The requirement for written informed consent was waived because the study used anonymised data. The Fire and Disaster Management Agency (FDMA) in Japan supervises the nationwide EMS system, while local fire stations operate the local EMS systems. As of 2017, the country has 732 fire departments and 5140 ambulance teams.^14^ During the study period, all EMS personnel performed CPR according to Japanese guidelines.^7^, ^8^, ^15^ Moreover, emergency lifesaving technicians who were EMS personnel used several other resuscitation techniques, including automated external defibrillators, airway adjuncts, peripheral intravenous catheters, and administration of Ringer’s lactate solution.^14^ In the field, only specially trained emergency lifesaving technicians, upon receiving instructions from an online physician, are permitted to insert a tracheal tube and administer intravenous adrenaline (epinephrine).^14^ EMS personnel in Japan are legally prohibited from terminating resuscitation in the field, except in specific situations such as decapitation, incineration, decomposition, rigor mortis, or dependent cyanosis. Therefore, most patients with OHCA receive CPR from EMS personnel before being transported to a hospital. Since 2006, emergency telephone dispatchers in Japan are required to provide instructions on how to perform chest-compression-only CPR if it is difficult for bystanders to administer rescue breathing.^5^

### Data collection and quality control

In 2005, the FDMA launched an ongoing, prospective, population-based observational study involving patients with OHCA who had received resuscitation from EMS personnel in Japan.^14^ Since 2005, with the co-operation of the physician-in-charge, EMS personnel from each centre have recorded the data of patients with OHCA using a Utstein-style template.^16^, ^17^ These data are transferred to local fire stations and subsequently integrated into the registry on the FDMA database server. The database software program automatically checks the data for consistency that is in turn verified by the FDMA. All the data are transferred and stored in the nationwide database developed by the FDMA for public use. The FDMA granted us permission to access the database and provided anonymous data for our analysis. The main variables included in the dataset were sex, age, aetiology of arrest, initially identified cardiac rhythm, bystander-witnessed status, type of witness (family member, layperson other than a family member, EMS personnel), type of CPR, time of collapse recognition, time of emergency call, time of vehicle arrival at the scene, time of CPR initiation by EMS, 1-month survival, and neurological outcomes 1 month after cardiac arrest. The aetiology of arrest was presumed to be cardiac unless evidence suggested traumatic causes (i.e., injury from a traffic accident, fall, accidental hypothermia, hanging, drowning, drug overdose/poisoning, or asphyxia) or other non-cardiac causes such as respiratory disease, cerebrovascular disease, or malignant tumours. The Physicians in charge and EMS personnel attempted to determine the origin of the arrest. Neurological outcomes were defined using the Cerebral Performance Category (CPC) scale (category 1, good cerebral performance; category 2, moderate cerebral disability; category 3, severe cerebral disability; category 4, coma or vegetative state; and category 5, death).^16^ CPC categorisation was determined by the physician-in-charge one month (30 days) after cardiac arrest. Information on bystander interventions and dispatchers providing CPR instruction was obtained by EMS personnel, who interviewed the bystanders before leaving the scene. All data were electronically recorded by EMS personnel and/or the EMS centre.

### Study endpoints

The primary outcome measure was 1-month neurologically intact survival, defined as a CPC score of 1 or 2. The secondary outcome measure was 1-month survival after OHCA.

### Statistical analysis

Continuous variables are expressed as medians and interquartile ranges, or as means and standard deviations, whereas categorical variables are expressed as percentages. To reduce the bias derived from differences in CPR strategies used over time, the study periods were divided into three groups based on the year that the major CPR guidelines were released: 2005–2010, 2011–2015, and 2016–2017. To investigate the trends of outcomes in some subgroups, the patients were divided into subgroups according to the type of CPR (no CPR, standard CPR, and chest-compression-only CPR), aetiology (traumatic or non-traumatic, with cardiac and non-cardiac subgroups in the non-traumatic group) and initial rhythm (shockable or non-shockable rhythm). We used the Kruskal–Wallis test followed by Dunn’s post hoc test to analyse continuous variables. The chi-square test and univariate logistic regression analyses were performed to compare the characteristics and outcomes of categorical variables. The Cochran–Armitage trend test was used to evaluate the trends in baseline characteristics and outcomes in the three study periods. To perform a rigorous adjustment of selection bias and thus determine the differences in the patients’ baseline characteristics, multivariate logistic regression analyses were used when comparing outcomes between groups and estimating the key prehospital variables associated with the outcomes. The potential prehospital confounders for the analytical model were selected based on biological plausibility and data from previous studies. In multivariate logistic regression analyses, eight prehospital variables were included: calendar year, age, sex, initial cardiac rhythm, cause of arrest, public-access defibrillation (PAD), bystander CPR status, and EMS response time (time from receipt of emergency call to EMS arrival at the patient’s location). All statistical analyses were performed using the JMP statistical package, version 15-Pro (SAS Institute Inc., Cary, NC, USA). All reported tests were two-tailed, and a *P*-value of < 0.05 was considered significant.

## Results

**Fig. 1:**
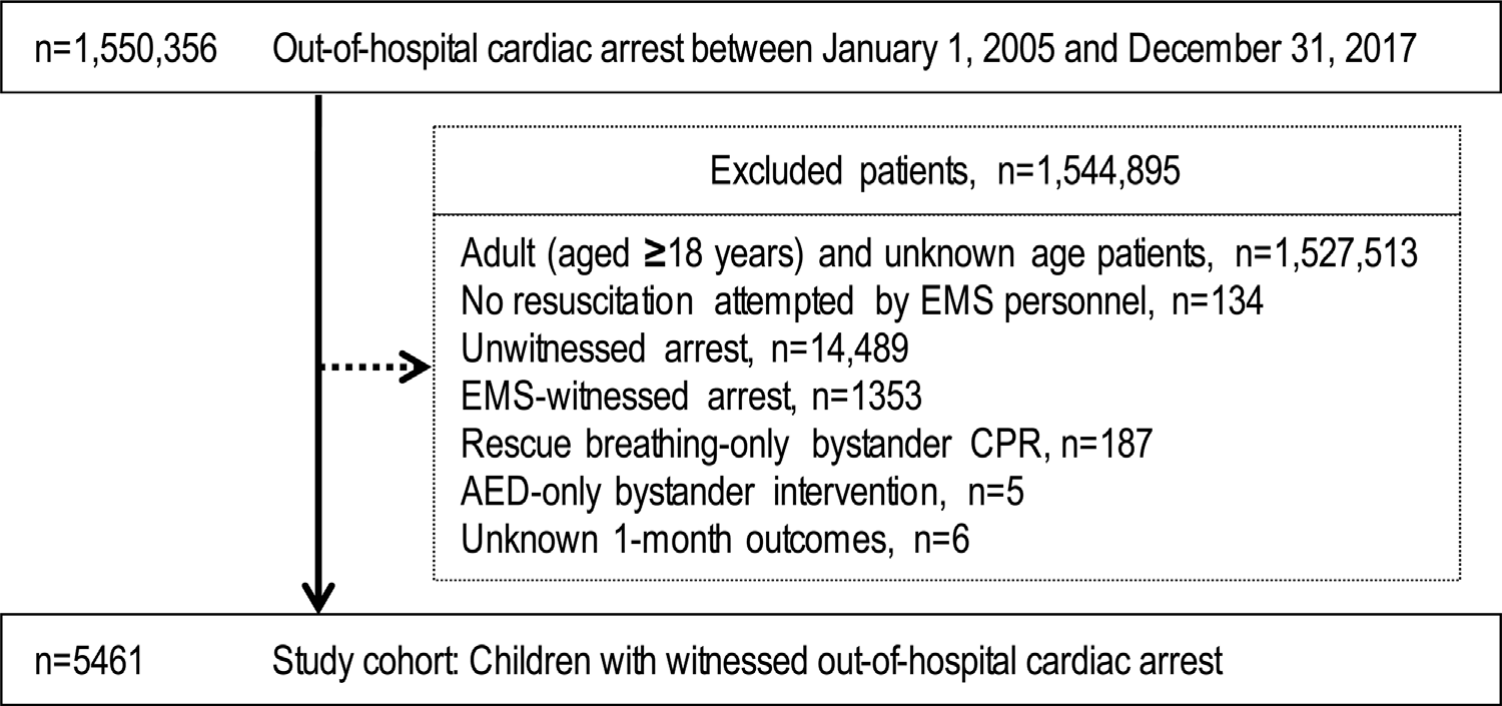
Flowchart of the study criteria. AED: automated electrical defibrillator, CPR: cardiopulmonary resuscitation, EMS: emergency medical services.

Details of attempted resuscitations performed for 1,550,356 patients with OHCA between 2005 and 2017 are documented in the database. Fig. 1 lists the inclusion and exclusion criteria of the study. Ultimately, 5461 paediatric patients (0.35% of registered patients) with witnessed OHCA were eligible.

Table 1 shows the baseline characteristics of the patients in the three study periods. The proportion of patients aged < 1 year significantly decreased over time, as did the proportion of patients who did not receive CPR and those who received standard CPR after the dispatcher offered CPR instructions. Increasing trends were observed in the rates of PAD, non-traumatic aetiology, dispatchers providing CPR instructions, overall bystander CPR, chest-compression-only CPR, and dispatcher-assisted CPR.

**Table 1:** Baseline characteristics of the participants in the three study periods.

			Overall	2005–2010	2011–2015	2016–2017	P-value for trend
Patients, n			5461	2686	2008	767	
Age, year							
	Median (IQR)		5 (0–14)	5 (0–14)	6 (1–14)	6 (1–14)	<0.05*
	Average (SD)		7.0 (6.5)	6.8 (6.5)	7.2 (6.5)	7.2 (6.5)	
	<1 year, n (%)		1406 (25.8)	742 (27.6)	492 (24.5)	172 (22.4)	<0.01
	1–11 years, n (%)		2230 (40.8)	1077 (40.1)	821 (40.9)	332 (43.2)	0.14
	12–17 years, n (%)		1825 (33.4)	867 (32.3)	695 (34.6)	263 (34.2)	0.14
Male, n (%)			3429 (62.8)	1684 (62.7)	1264 (63.0)	481 (62.7)	0.94
Initial shockable rhythm, n (%)			617 (11.3)	314 (11.7)	208 (10.4)	95 (12.4)	0.90
PAD, n (%)			172 (3.2)	61 (2.3)	74 (3.7)	37 (4.8)	<0.001
AED use by EMS personnel, n (%)			690 (12.6)	345 (12.8)	237 (11.8)	108 (14.1)	0.75
Non-traumatic aetiology, n (%)			3442 (63.0)	1659 (61.8)	1261 (62.8)	522 (68.1)	<0.01
	Cardiac, n/total n (%)		1877 (54,5)	910 (54.9)	677 (53.7)	290 (55.6)	0.99
	Non-cardiac, n/total n (%)		1565 (45.5)	749 (45.1)	584 (46.3)	232 (44.4)	0.99
EMS response time, min, n = 5456							
	Median (IQR)		8 (6–10)	8 (6–10)	8 (6–10)	8 (7–11)	<0.001*
	Average (SD)		9.0 (5.5)	8.6 (5.3)	9.3 (5.7)	9.6 (5.6)	
No-flow time, min, n = 5454							
	Median (IQR)		9 (6–14)	9 (6–13)	9 (6–14)	9 (6–14)	0.53*
	Average (SD)		11.3 (8.8)	11.3 (8.9)	11.3 (8.6)	11.6 (9.0)	
Dispatchers providing CPR instructions, n (%)			2198 (40.2)	941 (35.0)	904 (45.0)	353 (46.0)	<0.001
	Bystander response						
		No CPR, n (%)	521 (23.7)	247 (26.2)	214 (23.7)	60 (17.0)	<0.001
		Standard CPR, n (%)	630 (28.7)	357 (37.9)	196 (21.7)	77 (21.8)	<0.001
		Chest-compression-only CPR, n (%)	1047 (47.6)	337 (35.8)	494 (54.6)	216 (61.2)	<0.001
Bystander CPR, n (%)			2779 (50.9)	1286 (47.9)	1053 (52.4)	440 (57.4)	<0.001
	Type of bystander CPR manoeuvre						
		Standard CPR, n (%)	1177 (42.4)	707 (55.0)	340 (32.3)	130 (29.6)	<0.001
		Chest-compression-only CPR, n (%)	1602 (57.6)	579 (45.0)	713 (67.7)	310 (70.4)	<0.001
	Dispatcher-assisted CPR, n (%)		1677 (60.3)	694 (54.0)	690 (65.5)	293 (66.6)	<0.001

AED, automated external defibrillator; CPR, cardiopulmonary resuscitation; CPC, Cerebral Performance Category; EMS, emergency medical services; EMS response time, time from the emergency call receipt to EMS arrival at the patient’s site; IQR, interquartile range; No-flow time, time from collapse to initiation of CPR by bystanders or EMS providers; PAD, public-access defibrillation; SD, standard deviation.

* P-value for Wilcoxon test.

**Fig. 2:**
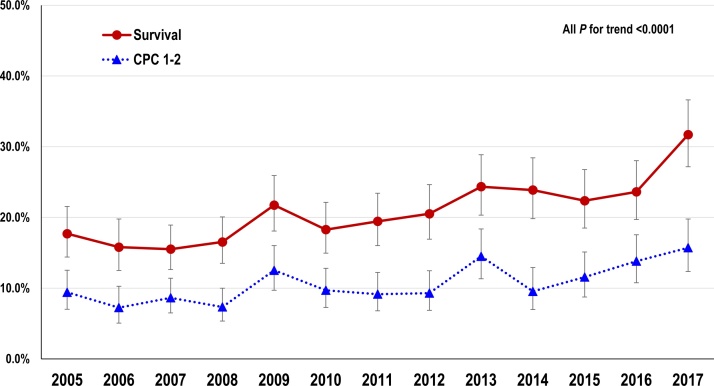
Crude 1-month outcomes by year. CPC: Cerebral Performance Category.

The crude 1-month survival and CPC 1–2 rates significantly increased by year (Fig. 2, all P for trend < 0.001): survival = from 17.7% in 2005 to 31.7% in 2017 and CPC 1–2 = from 9.4% in 2005 to 15.7% in 2017. The temporal trends in the crude 1-month outcomes by three study periods are shown in Table 2. Survival and CPC 1–2 rates significantly increased over time in all patients (both P for trend < 0.001). By type of CPR, similar improvements in survival and CPC 1–2 rates were observed with both standard and chest-compression-only CPR groups (P for trend < 0.05 and < 0.001 for survival rate and < 0.01 and < 0.001 for CPC 1–2 rate, respectively). In the subgroup analyses by aetiology, the survival and CPC 1–2 rates in patients with OHCA of non-traumatic origin significantly increased (both P for trend < 0.001) but not in those with OHCA of traumatic origin (P for trend = 0.87 and 0.29, respectively). In the subgroup analyses by initial rhythm, the survival and CPC 1–2 rates significantly improved regardless of rhythm stratification (all P for trend < 0.001).

**Table 2:** Temporal trends in the crude 1-month outcomes.

				Overall	2005–2010	2011–2015	2016–2017	P-value for trend
Overall, n				5461	2686	2008	767	
			Survival, n (%)	1124 (20.6)	471 (17.5)	442 (22.0)	211 (27.5)	<0.001
			CPC 1 or 2, n (%)	574 (10.5)	245 (9.1)	216 (10.8)	113 (14.7)	<0.001
Type of bystander CPR								
	No CPR, n			2682	1400	955	327	
			Survival, n (%)	409 (15.3)	182 (13.0)	170 (17.8)	57 (17.4)	<0.01
			CPC 1 or 2, n (%)	172 (6.4)	80 (5.7)	68 (7.1)	24 (7.3)	0.15
	Standard CPR, n			1177	707	340	130	
			Survival, n (%)	336 (28.6)	184 (26.0)	101 (29.7)	51 (39.2)	<0.01
			CPC 1 or 2, n (%)	202 (17.2)	110 (15.6)	62 (18.2)	30 (23.1)	<0.05
	Chest-compression-only CPR, n			1602	579	713	310	
			Survival, n (%)	379 (23.7)	105 (18.1)	171 (24.0)	103 (33.2)	<0.001
			CPC 1 or 2, n (%)	200 (12.5)	55 (9.5)	86 (12.1)	59 (19.0)	<0.001
Aetiology								
	Traumatic origin, n			2019	1027	747	245	
			Survival, n (%)	234 (11.6)	121 (11.8)	81 (10.8)	32 (13.1)	0.87
			CPC 1 or 2, n (%)	86 (4.3)	50 (4.9)	26 (3.5)	10 (4.1)	0.29
	Non-traumatic origin, n			3442	1659	1261	522	
			Survival, n (%)	890 (25.9)	350 (21.1)	361 (28.6)	179 (34.3)	<0.001
			CPC 1 or 2, n (%)	488 (14.2)	195 (11.8)	190 (15.1)	103 (19.7)	<0.001
		Cardiac, n		1877	910	677	290	
			Survival, n (%)	519 (27.7)	218 (24.0)	194 (28.7)	107 (36.9)	<0.001
			CPC 1 or 2, n (%)	334 (17.8)	134 (14.7)	128 (18.9)	72 (24.8)	<0.001
		Non-cardiac, n		1565	749	584	232	
			Survival, n (%)	371 (23.7)	132 (17.6)	167 (28.6)	72 (31.0)	<0.001
			CPC 1 or 2, n (%)	154 (9.8)	61 (8.1)	62 (10.6)	31 (13.4)	<0.05
Initial rhythm								
	Shockable rhythm, n			617	314	208	95	
			Survival, n (%)	303 (49.1)	132 (42.0)	104 (50.0)	67 (70.5)	<0.001
			CPC 1 or 2, n (%)	236 (38.2)	98 (31.2)	88 (42.3)	50 (52.6)	<0.001
	Non-shockable rhythm, n			4844	2372	1800	672	
			Survival, n (%)	821 (16.9)	339 (14.3)	338 (18.8)	144 (21.4)	<0.001
			CPC 1 or 2, n (%)	338 (7.0)	147 (6.2)	128 (7.1)	63 (9.4)	<0.001

CPR, cardiopulmonary resuscitation; CPC, Cerebral Performance Category.

Multivariate logistic regression analyses demonstrated that the 2016–2017 period and standard CPR were independently associated with increased odds of favourable 1-month outcomes compared with the other time periods or CPR groups, respectively (Table 3). Initial shockable rhythm, PAD and non-traumatic aetiology were also associated with favourable outcomes. On calculating adjusted outcomes rates using the adjusted odds ratio of each period compared with the 2005–2010 period (17.5% for survival rate, 9.1% for CPC 1–2 rate), the adjusted outcomes rates were as follows: survival rate = 25.6% (2011–2015) and 33.1% (2016–2017) and CPC 1–2 rate = 12.3% (2011–2015) and 16.9% (2016–2017).

**Table 3:** Adjusted odds ratios of prehospital variables for 1-month outcomes in overall patients (n = 5456).

Variables		Adjusted OR (95% CI)			
		1-month survival		1-month CPC 1 or 2	
Year					
	2011–2015 (vs. 2005–2010)	1.46	(1.25–1.71)	1.35	(1.09–1.68)
	2016–2017 (vs. 2005–2010)	1.89	(1.54–2.32)	1.86	(1.41–2.44)
	2016–2017 (vs. 2011–2015)	1.29	(1.06–1.59)	1.37	(1.04–1.81)
Age^a^		0.99	(0.98–1.00)	1.03	(1.01–1.05)
Male (vs. female)		1.00	(0.87–1.16)	0.93	(0.76–1.14)
Initial shockable rhythm (vs. initial non-shockable rhythm)		3.60	(2.95–4.38)	4.92	(3.92–6.18)
PAD (vs. no PAD)		3.90	(2.72–5.60)	4.51	(3.09–6.59)
Non-traumatic aetiology (vs. traumatic aetiology)		1.73	(1.46–2.06)	2.21	(1.70–2.88)
Type of bystander CPR					
	Standard CPR (vs. no CPR)	1.60	(1.33–1.92)	1.86	(1.45–2.39)
	Standard CPR (vs. chest-compression-only CPR)	1.28	(1.06–1.54)	1.38	(1.08–1.76)
	Chest-compression-only CPR (vs. no CPR)	1.25	(1.06–1.48)	1.34	(1.06–1.71)
EMS response time^a^		0.93	(0.91–0.95)	0.89	(0.86–0.92)

CPR, cardiopulmonary resuscitation; CI, confidence interval; CPC, Cerebral Performance Category; EMS, emergency medical services; OR, odds ratio; PAD, public-access defibrillation.

a Adjusted odds ratios are reported for 1-year or 1-min increments.

## Discussion

This nationwide, population-based observational study in Japan demonstrated that the 1-month survival and CPC 1–2 rates of paediatric patients with bystander-witnessed OHCA significantly improved during three study periods, with an adjusted odds ratio (95% confidence interval) of 1.89 (1.54–2.32) and 1.86 (1.41–2.44), respectively. A similar trend was observed in the 1-month outcomes of some subgroups: those who received CPR (both standard CPR and chest-compression-only CPR) and those with OHCA of non-traumatic origin (both cardiac and non-cardiac). Even after adjusting for confounding factors, both 1-month survival and neurologically intact survival in the 2016–2017 period were twice of those in the 2005–2010 period. To the best of our knowledge, this is the first and largest cohort study to show that 1-month survival and neurologically intact survival increased over time in Japanese paediatric patients with OHCA.

There are many possible reasons for the improvement in outcomes during the study period. In accordance with the 2005 CPR guidelines,^15^, ^18^ emergency dispatch centres in Japan have become increasingly active in relaying CPR instructions to citizens performing CPR.^5^ In 2006, dispatcher-assisted CPR instruction was converted from instructions regarding standard CPR with rescue breaths to chest-compression-only CPR for both adult and paediatric patients if bystanders experienced difficulties providing rescue breaths.^5^ The nationwide dissemination of the following recommendations based on the 2010 and 2015 CPR guideline updates may have contributed to the improvement in patient outcomes^7^, ^8^: (1) high-quality CPR assisted by a dispatcher on the phone; (2) change from the ‘airway-breathing-circulation’ to ‘circulation-airway-breathing’ sequence for CPR; (3) improvement in post-resuscitation care (e.g., targeted temperature management). In 2017, the local fire department in Japan trained approximately 1.3 million citizens using conventional 3-h CPR programmes comprising chest compressions, mouth-to-mouth ventilation, and AED.^14^ The rate of chest-compression-only CPR during dispatcher-assisted CPR increased from 49% in 2005–2010 to 74% in 2016–2017. Moreover, the PAD rates doubled (from 2.3%–4.8%). Because of the abovementioned efforts in Japan, early CPR by bystanders and PAD may have contributed to the improvement in outcomes in children who experienced OHCA. A decrease in the proportion of patients aged < 1 year and those with traumatic aetiology may also have contributed to the increase in 1-month favourable outcomes after OHCA. Unfortunately, no increase in the rate of initial shockable rhythm or AED use by EMS personnel was observed during the study period neither was there a decrease in the duration of no-flow time or EMS response time.

Regarding the CPR type, 1-month outcomes of both the standard CPR and chest-compression-only CPR cohorts improved over time. Although there may be a number of things contributing to this improvement that are clearly not explained by CPR, the present study supports the 2017 International Liaison Committee on Resuscitation summary statement^3^ and the European Resuscitation Council 2017 guideline update^4^: It is better to provide rescue breaths as part of the resuscitation sequence for children; if bystanders cannot provide rescue breaths, they should at least provide chest compressions.

A previous study showed that survival rates in paediatric patients who experienced in-hospital cardiac arrest in US significantly improved between 2000 and 2018.^11^ However, few previous studies have reported temporal trends in survival and neurologically intact survival in paediatric patients with OHCA. More recently, in Australia, Nehme et al. showed that the rate of survival to hospital discharge following non-traumatic paediatric OHCA increased from 2000 to 2016, consistent with the results of the present study.^13^ We also demonstrated an improvement in 1-month neurologically intact survival among children with non-traumatic OHCA. Jayaram et al. found no improvements in survival in children with OHCA of cardiac origin over the following three time periods in the US: 2005–2007, 2008–2010, and 2011–2013.^12^ This result was inconsistent with our study, and there are several potential reasons for this. First, the characteristics of the patients differed between the two studies. The aforementioned study only focused on OHCA patients with a cardiac aetiology, regardless of whether any witnesses were present. We included patients with bystander-witnessed OHCA, regardless of aetiology. Second, we analysed Japanese nationwide data over 13 years, including those recorded after the 2015 guideline update, while Jayaram et al. analysed data from the Cardiac Arrest Registry to Enhance Survival for 9 years that did not include data collected after the 2015 guideline update. Therefore, the results may have differed because the study periods differed and strategies for managing paediatric OHCA improved after the update in the 2015 international CPR guidelines. Lastly, the inherent differences between two studies in EMS systems and post-cardiac arrest care may be attributable to these differences.

The increase in the rate of dispatcher-provided CPR instructions in cases of paediatric patients with OHCA may have improved overall outcomes because this rate significantly increased from 35% to 46% over time in this study. The proportion of patients who received CPR following dispatcher’s instructions also increased—from 74% to 83%. However, 17% of bystanders did not perform CPR despite the dispatcher’s offer. Accordingly, to improve neurological outcomes, every effort should be made to reduce the proportion of non-responders. Furthermore, to ensure that dispatcher-assisted CPR is administered, it is essential that dispatchers recognise cardiac arrest during an emergency call.^19^, ^20^ Therefore, efforts should focus on increasing the early recognition of cardiac arrest so that CPR instructions can be offered by the dispatcher to bystanders. A recent Parisian study^21^ reported a unique method called the ‘hand-on-belly’ method to detect OHCA based on details provided by a caller over the phone. This novel method significantly increased the detection of OHCA and dispatcher-assisted CPR to 83% and 93% in 2018, respectively, achieving the goals recommended by the American Heart Association.^22^ This new method of recognising cardiac arrest over the phone may help increase the early recognition of cardiac arrest in paediatric patients.

This study has some limitations. First, we stratified the study periods into three unequal groups according to the release of major CPR guidelines to reduce the bias derived from differences in CPR strategies used over time. However, this may have led to the inclusion of unexpected confounders. Second, the actual aetiology of cardiac arrest was not fully verified. Therefore, some patients may have had sudden infant death syndrome, a common aetiology of cardiac arrest, followed by trauma and respiratory disease.^23^ Third, although we used a uniform data collection procedure, a large sample size, and a population-based design, we cannot exclude the possibility of uncontrolled confounders, such as pre-existing comorbidities, location of the arrest, quality of bystander-initiated CPR, and in-hospital treatments because the study was retrospective and observational. Therefore, we could not include these data in our analyses. Forth, as with all epidemiological studies, selection bias may have occurred, and the data may have lacked integrity and validity. Finally, the relevance of our results to other communities that have different emergency care systems and protocols remains unknown. Therefore, similar analyses in other countries are required to validate our results.

## Conclusion

Between 2005 and 2017 in Japan, the 1-month neurologically intact survival rate of paediatric OHCAs significantly increased in patients with bystander-witnessed OHCA of non-traumatic origin, regardless of the CPR type. No such improvement was seen in paediatric patients with bystander-witnessed OHCA of traumatic origin.

## Funding sources

This work was supported by the Japan Society for the Promotion of Science(Grant-in-Aid for Scientific Research: Grant Nos. 20H202271 and18K09999).

## Authors’ contributions

Yoshikazu Goto and Akira Funada designed the study. Yoshikazu Goto, Akira Funada, Tetsuo Maeda, and Yumiko Goto sorted the data. Yoshikazu Goto and Yumiko Goto analysed the data. Yoshikazu Goto drafted the manuscript, and Yumiko Goto and Akira Funada substantially contributed to its revision. Yoshikazu Goto takes responsibility for the paper as a whole. All authors read and approved the final manuscript.

## Conflicts of interest

None.

## Data statement

The datasets generated during and/or analysed during the current study are not publicly available because of the FDMA regulations but are available from the corresponding author on reasonable request.
